# Genetic Predisposition to Neuroblastoma

**DOI:** 10.3390/children5090119

**Published:** 2018-08-31

**Authors:** Erin K. Barr, Mark A. Applebaum

**Affiliations:** 1 Department of Pediatrics, University of Chicago, Chicago, IL 60637, USA; ebarr1@peds.bsd.uchicago.edu; 2 Committee on Clinical Pharmacology and Pharmacogenomics, University of Chicago, Chicago, IL 60637, USA

**Keywords:** neuroblastoma, predisposition, germline, genome-wide association study (GWAS)

## Abstract

Neuroblastoma is the most common solid tumor in children under the age of one. It displays remarkable phenotypic heterogeneity, resulting in differences in outcomes that correlate with clinical and biologic features at diagnosis. While neuroblastoma accounts for approximately 5% of all cancer diagnoses in pediatrics, it disproportionately results in about 9% of all childhood deaths. Research advances over the decades have led to an improved understanding of neuroblastoma biology. However, the initiating events that lead to the development of neuroblastoma remain to be fully elucidated. It has only been recently that advances in genetics and genomics have allowed researchers to unravel the predisposing factors enabling the development of neuroblastoma and fully appreciate the interplay between the genetics of tumor and host. In this review, we outline the current understanding of familial neuroblastoma and highlight germline variations that predispose children to sporadic disease. We also discuss promising future directions in neuroblastoma genomic research and potential clinical applications for these advances.

## 1. Introduction

Neuroblastoma is the most common tumor seen in the first year of life, and is the most frequent extracranial solid tumor of childhood [1]. It is derived from neural crest cells, and may arise in the adrenal medulla or anywhere along the paraspinal sympathetic ganglia [1]. Typically, neuroblastoma presents with bone pain, anemia, or, in babies, hepatomegaly, and has a median age at diagnosis of 18 months [1]. While neuroblastoma accounts for approximately 5% of all cancer diagnoses in pediatrics, it disproportionately results in about 9% of all childhood deaths from cancer [2].

Compared to other adult and pediatric malignancies, neuroblastoma has remarkable phenotypic heterogeneity, ranging from spontaneous regression with no treatment, to relentless progressive disease resistant, to intensive multimodal therapy [3,4,5]. Approximately 40% of children diagnosed with neuroblastoma are classified as high-risk, and only 50% of these patients will achieve long-term survival [4,6]. Conversely, patients with low- and intermediate-risk disease do quite well, achieving greater than 95% overall survival [4]. One consequence of this phenotypic heterogeneity has been the considerable effort placed on prognosticating outcome at the time of diagnosis to ensure that treatment is optimally tailored. The International Neuroblastoma Risk Group (INRG) classification system uses seven clinical and biologic factors associated with outcome at diagnosis to classify neuroblastoma tumors into four categories (very low-risk, low-risk, intermediate-risk, and high-risk) [6,7]. The expectation of this nonsurgical classification system is to allow for comparison of patient outcomes by risk group across international cooperative groups. 

**Figure 1:**
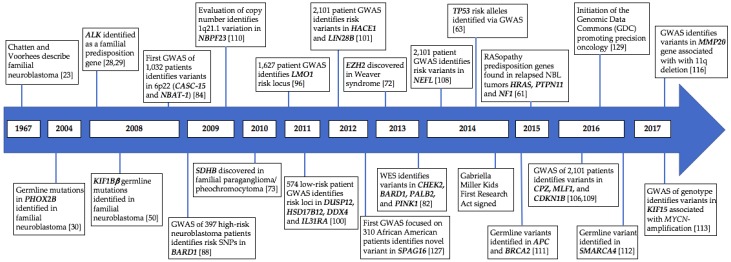
Timeline of identified genetic variation predisposing to neuroblastoma. GWAS, genome-wide association studies.

Despite the progress made in the treatment and risk stratification of neuroblastoma patients, it is only recently that the field has begun to appreciate the interplay between the genetics of tumor and host. Somatic genetic aberrations were identified more than three decades ago with the discovery of the prognostic significance of *MYCN* status and tumor ploidy [8]. The link between *MYCN*-amplification and aggressive disease remains one of the most significant components of risk stratification and treatment selection [6,9,10,11,12]. The importance of DNA ploidy was first described in 1984 [13] and while diploid and hypodiploid status is associated with *MYCN*-amplification, this genetic feature confers additional poor prognosis [1,8,14]. The late 1980s and 1990s saw the discovery of segmental chromosomal aberrations in 1p, 11q, and 17q as additional genetic markers of worse outcome [15,16,17]. Over the past 15 years, additional genomic features including chromosome copy number, transcriptomics, and epigenetics have all proven to have a role in neuroblastoma pathogenesis [18,19,20,21,22]. While these advances have allowed for an improved understanding of neuroblastoma biology, they are unable to answer a most basic question asked by patients and families: Why does my child have neuroblastoma? It has only been in the past ten years that advances in genetics have allowed researchers to begin to address this question in earnest. In this review, we will outline the current understanding of the genetics of familial neuroblastoma and will also highlight recent germline variations that predispose children to sporadic disease (Figure 1). In addition, we will discuss exciting future directions of genomic research in neuroblastoma and potential clinical applications for these advances.

## 2. Familial Neuroblastoma

Though the majority of neuroblastoma tumors arise sporadically, rarely this cancer is heritable. In 1967, Chatten and Voorhess were the first to recognize this heritability in their description of a family in which four out of five siblings developed early onset neuroblastoma [23]. Additional case reports of families with an unusually high incidence of the disease were described subsequently [24,25]. We now know that familial neuroblastoma occurs in approximately 1–2% of neuroblastoma cases [1]. Familial neuroblastoma tends to present at a younger age, with a family history of relatives with the same tumor, and an increased likelihood of multiple primary sites, particularly tumors involving both adrenal glands [26]. It is usually inherited in an autosomal dominant manner, with incomplete penetrance and, similar to retinoblastoma, conforms to the classic two-hit model [26,27]. Additionally, some family members harboring predisposing genetic mutations do not develop neuroblastoma [28,29]. Although familial neuroblastoma is rare, careful investigation of these cases has yielded important information on neuroblastoma tumorigenesis and identified avenues for novel targeted therapies.

### 2.1. PHOX2B

While the existence of familial neuroblastoma had been described for decades, it was not until 2004 that the first neuroblastoma predisposition gene, paired-like homeobox 2B gene (*PHOX2B*), was identified [30,31]. It was noted that patients with diseases of neural crest origin, such as congenital central hypoventilation syndrome (CCHS) and Hirschsprung disease, had a 5–10% chance of developing neuroblastic tumors, compared to 0.01% for the general population [32]. *PHOX2B* was first identified as a driver of these developmental disorders, and later identified in neuroblastoma patients with a family history of neuroblastic tumors or congenital malformations [30]. These insights culminated in the recognition that mutations in *PHOX2B* predispose children to familial neuroblastoma [30,31,33,34].

The *PHOX2B* gene regulates autonomic nervous system development [35] and, in children with CCHS, there is a link between specific mutations and neuroblastic tumor development [33,36]. Children with *PHOX2B* frameshift or missense mutations (nonpolyalanine repeat mutations) are more likely to develop more severe disease with central nervous system tumors, and Hirschsprung’s disease, than those with the more common polyalanine repeat expansion mutations (PARM) suggesting that these mutations may impart more severe disruption to gene function [33,36,37]. Both Trochet and Mosse took blood from families with familial neuroblastoma and sequenced the *PHOX2B* gene discovering mutations in *PHOX2B* that passed from parents to their children [30,34]. Of note, not all families with neuroblastoma had a *PHOX2B* mutation, suggesting that other predisposition genes were yet to be discovered [34], and we now know that about six to ten percent of familial neuroblastoma cases will have a mutation in *PHOX2B* [34,38]. Additionally, PHOX2B mutations also occur in about 2% of sporadic cases of neuroblastoma [39,40].

### 2.2. ALK

The anaplastic lymphoma kinase (*ALK*) gene was identified as a neuroblastoma predisposition gene in 2008 in two studies; one, a genetic linkage study across 20 families with neuroblastoma and the other, a genome-wide comparative genomic hybridization analysis of 592 neuroblastoma tumors [28,29]. Three germline missense mutations (R1192P, R1275Q, and G1128A), were identified in the tyrosine kinase domain of *ALK* that were present in the majority of familial neuroblastoma cases evaluated. Concurrently, mutations in *ALK* were also reported in 10–12% of sporadic cases of neuroblastoma [28,29,41,42]. In neuroblastoma, constitutive activation of the tyrosine kinase domain of *ALK* by multiple possible genetic mechanisms results in increased oncogenicity [28,43,44]. Linking both *PHOX2B* and *ALK* biology, some neuroblastoma cell lines, inducing overexpression of *PHOX2B*, led to increased *ALK* expression [45]. Germline *ALK* mutations have incomplete phenotypic penetrance, meaning that not all affected individuals will develop neuroblastoma [43]. As has been described [46], different mutations in *ALK* confer a specific susceptibility to targeted inhibitors such as crizotinib. The R1275Q mutation is found in both familial and sporadic tumors and has higher penetrance than the weaker activating mutation G1128A [28,47]. Cell lines with R1275Q mutations also have greater sensitivity to ALK inhibition when treated with the small molecule inhibitor of *ALK,* crizotinib, than those with the common somatic mutation found at F1174L [48]. The presence of *ALK* mutations, initially identified through the small number of familial neuroblastoma cases, has now become the basis for treatment stratification in the recently opened Children’s Oncology Group (COG) study ANBL1531 which integrates crizotinib into the upfront setting for individuals with *ALK*+ disease (NCT03126916).

### 2.3. KIF1Bβ

*KIF1Bβ* is a proposed tumor suppressor gene thought to be involved in the pathogenesis of neural crest tumors such as neuroblastoma and pheochromocytoma [49]. Germline mutations in this gene are proposed to provide a survival advantage to neuronal progenitor cells with malignant potential, ultimately allowing them to develop into an aggressive tumor [49]. Yeh et al. evaluated five related individuals who had both neural crest-derived tumors (neuroblastoma, ganglioneuroma, and pheochromocytomas) and non-neural crest-derived tumors (leiomyosarcoma and lung adenocarcinoma). *KIF1Bβ* germline mutations were found in individuals who developed neuronal tumors. Furthermore, these patients had germline findings consistent with known somatic neuroblastoma biology with haploinsufficiency or methylation of the wild-type allele of *KIF1Bβ*. Conversely, in a patient with lung adenocarcinoma, there was loss of the wild-type allele, consistent with classic two-hit inactivation. These results suggest a gene dosage effect for tumor development with different tissues requiring only mono-allelic inactivation, while others require bi-allelic inactivation of the *KIF1Bβ* gene [50].

### 2.4. RAS Pathway Mutations and Other Cancer Predisposition Syndromes

While the genes described above were identified in families with a predisposition specifically for neuroblastic tumors, those with other cancer predisposition syndromes can be at increased risk of developing neuroblastoma in addition to other malignancies. These syndromes primarily involve genes in the canonical RAS pathway including Costello syndrome, Noonan syndrome, and neurofibromatosis type 1 [32,51,52,53,54,55,56,57,58,59]. RAS proteins control intracellular growth, differentiation, and survival signaling, including the important downstream mitogen-activated protein (MAP) kinase pathway. Mutations that disrupt any of the key proteins in this important pathway may result in a RASopathy [60]. Neural crest cells, from which neuroblastoma originates, migrate and innervate multiple organs and rely on RAS signaling for terminal maturation. Failure of neural crest cells to develop correctly, due to RAS pathway mutations, may help explain the prevalence of neuroblastoma in these predisposition syndromes. Confirming the importance of the RAS-MAPK pathway in neuroblastoma is the observation that recurrent, sporadic tumors have high frequencies of mutations in this pathway, and these mutations are an indicator of more aggressive disease [61].

### 2.5. Other Predisposition Syndromes

Recently, neuroblastoma has been described in patients with Li–Fraumeni syndrome and hereditary pheochromocytoma/paragangliomas syndromes [51]. In Li–Fraumeni, the R337H mutation in *TP53* appears to have a particularly strong association with the development of neuroblastoma [62,63]. ROHHAD syndrome (rapid-onset obesity, hypothalamic dysfunction, hypoventilation and autonomic dysfunction) is a rare disorder which is clinically similar to CCHS [64]. It is frequently associated with neural crest tumors, such as neuroblastoma, ganglioneuroma, or ganglioneuroblastoma; however, its genetic origins are yet to be elucidated [65,66,67]. Beckwith–Wiedemann syndrome is a syndrome of overgrowth, characterized by hemihypertrophy, macroglossia, midline abdominal wall defects, and macrosomia. It is caused by abnormal methylation of chromosome 11p15, or uniparental disomy of that region, which disrupts the expression of *CKDN1C*, an inhibitor of cell proliferation. Patients with this syndrome are predisposed to developing hepatoblastoma and Wilms tumor, but also have an increased incidence of neuroblastoma (2–5% risk) [68,69,70,71]. Weaver syndrome and familial paraganglioma/ pheochromocytoma have also been linked to neuroblastoma development, with mutations found in *EZH2* and *SDHB*, respectively [72,73,74]. Fanconi anemia, a rare chromosomal instability disorder, has been associated with neuroblastoma in addition to many other cancers [75,76,77]. Although there are many genetic mutations associated with Fanconi anemia, the most common mutations associated with cancer predisposition are *BRIP1*, *BRCA2,* and *PALB2* [75,78,79]. Though the exact excess risk of developing neuroblastoma has yet to be fully elucidated for these rare syndromes, a summary of known associations can be seen in Table 1. Additionally, the higher rates of neuroblastoma seen in some of these aforementioned genetic syndromes have been thought to warrant neuroblastoma surveillance, which has recently been outlined [51].

## 3. Sporadic Neuroblastoma

Though many cancers can be attributed to genetic changes induced by hereditary predisposition, as described in neuroblastoma for the *PHOX2B* and *ALK* genes, others may be the result of lifestyle or environmental factors. However, these modifiable factors are now thought to only account for about one-third of all cancers globally [80,81], and are not known to be contributory to the development of neuroblastoma. Additionally, perplexing in neuroblastoma tumorigenicity is the fact that only a small subset of tumors have an identifiable oncogenic driver mutation [82]. Together, these findings raise the fundamental question of why some children develop neuroblastoma, and what predisposing genetic factors may be present in these patients. Due to the inherent limitations of candidate gene and family association studies, this question could not be addressed until the development of genotyping arrays and high-performance computing allowed researchers to conduct Genome-wide association studies (GWAS) to identify germline genetic variation that may lead to development of neuroblastoma [83]. A summary of germline GWAS variants predisposing to neuroblastoma can be seen in Table 2.

### 3.1. Susceptibility to Sporadic Neuroblastoma

In 2008, the first successful GWAS in neuroblastoma compared 1032 neuroblastoma patients to 2043 healthy controls of European descent, using 464,934 single-nucleotide polymorphisms (SNPs) and a small replication cohort [84]. This study identified three variants in chromosome 6p22, which have been subsequently mapped to the genes *CASC-15* and *NBAT-1* (*CASC-14*), subsequently identified as long noncoding RNAs. The most statistically significant SNP was rs6939340, which was found to be more prevalent in patients who developed high-risk, *MYCN*-amplified, or stage 4 tumors. Subsequent studies established that this SNP is intronic to the long noncoding RNA *CASC-15,* and leads to a short isoform *CASC15-S* [85]. Additionally, expression of this isoform is decreased in high-risk tumors and patients with poor survival. Decreased expression of *NBAT-1* has also been correlated with high-risk neuroblastoma [86]. Ten years after the initial GWAS, we now have a better mechanistic understanding of this genetic predisposition as *CASC15* and *NBAT-1* were shown to modulate the localization of the Ubiquitin-Specific Protease 36 (*USP36*) [87]. When these two long noncoding RNAs are lost, CHD7 is de-ubiquitinated by USP36 and then interacts with SOX9 to maintain a de-differentiated cellular state. Additional studies are ongoing to determine how this novel system can be therapeutically disrupted with the goal of differentiating neuroblastoma cells into a benign state.

As the locus in 6p22 was enriched primarily in patients who developed high-risk tumors, the next analysis of the same data set restricted the discovery cohort to those 397 high-risk patients from the original 1032 patient cohort and compared them to the same 2043 controls [88]. In addition to confirming the 6p22 locus, several new risk SNPs were identified in chromosome 2q35 that were intronic to the *BARD1* gene. Moreover, these same risk SNPs have since been validated independently in an Italian cohort [89]. At the time of this discovery, it was recognized that BARD1 bound BRCA1 [90,91], though the role for *BARD1* as a cancer predisposition gene was previously unknown. Functional studies of *BARD1* demonstrated that a BARD1 isoform has high oncogenic activity and is sufficient for neoplastic transformation of mouse fibroblasts [92]. Furthermore, BARD1 has a mechanistic interaction with AURKA, which has an active role in stabilizing MYCN protein. In vitro analysis demonstrated that the AURKA is required for the growth of *MYCN*-amplified neuroblastoma cell lines [93] and the AURKA inhibitor alisertib (MLN8237) was able to inhibit the growth of neuroblastoma xenografts [94]. Unfortunately, while the drug was well tolerated, the objective response rate of 18.8% [95] was not sufficient for continued development of alisertib. This chain of research stemming from a GWAS result stands as clear proof of concept that GWAS can identify clinically actionable biology in neuroblastoma.

Over time, the cohort of genotyped neuroblastoma patients increased towards the ultimate goal of accruing 7500 affected children. Three years after the first analysis of this cohort, 1627 patients with any phenotype of neuroblastoma were compared to 3254 controls [96]. This study confirmed risk variants in *CASC15* and *BARD1,* and identified a new locus on 11p15.4 mapping to the *LMO1* gene. The LMO family of genes had been previously implicated in the development of leukemia and breast cancer [97], but this was the first time *LMO1* had been linked to neuroblastoma. The variant allele, rs110419, found in 55% of neuroblastoma patients compared to 45% in healthy controls, results in a gain of function mutation that increases *LMO1* expression [96]. In a zebrafish model of neuroblastoma, increased LMO1 synergizes with MYCN to promote tumorigenesis of aggressive phenotype neuroblastoma [98]. Further fine mapping and functional analysis then identified the protective variant SNP rs2168101 G > T to be more prevalent in healthy controls (31.3%) compared to neuroblastoma cases (24.2%, OR 0.65). This protective variant is located in a superenhancer element of *LMO1* and the nucleotide conversion alters a GATA binding site, preventing the binding of multiple transcription factors which would otherwise lead to a malignant expression pattern [99].

Using a similar approach to what had been previously reported for the subgroup of high-risk tumors, the 574 low-risk patients were extracted from the larger cohort of 1627 neuroblastoma patients, and compared to 1722 controls matched 3:1 [100]. This GWAS led to the identification of one susceptibility SNP in *DUSP12,* and three SNPs in *HSD17B12,* statistically associated with the development of low-risk neuroblastoma [100]. Additional testing identified *DDX4* and *IL31RA* as having interactions at the gene/variant level, suggesting that these variants also predispose children to low-risk neuroblastoma. While the mechanisms underlying these associations are not yet well understood, these findings again highlight the differing genetic underpinnings of high- and low-risk disease.

As the cohort of neuroblastoma genotyping grew, it became possible to identify variants with lower allele frequencies that were associated with the development of neuroblastoma. In 2012, Diskin et al. conducted a GWAS comparing a discovery cohort of 2101 patients, twice as many cases than four years prior, to 4202 European controls [101]. While confirming the previous findings already described, the investigators identified new risk variants in *HACE1* and *LIN28B* that were associated with the development of neuroblastoma. Of note, this was the first GWAS in neuroblastoma to evaluate an African American cohort, confirming predisposition SNPs in *HACE1* and identifying a trend towards significance for those in *LIN28B*. Additionally, even though it was in validation analysis, this was also the first neuroblastoma GWAS to impute SNPs based on phase 1 of the 1000 Genomes Project [102]. While *HACE1* has been documented as a tumor suppressor in many cancers including neuroblastoma [103], the exact function of this gene and the associated variant have yet to be fully elucidated. In contrast, *LIN28B* has been shown to be overexpressed in high-risk neuroblastoma, leading to increased *MYCN* expression and stabilization by both inhibiting the miRNA let-7, and increasing *RAN* and *AURKA* expression [104,105].

Subsequently, this set of 2101 Caucasian patients was again compared to the same controls [106]. Due to advances in imputation and the availability of the 1000 Genomes phase 1 v3 release [107], it was possible to evaluate almost eight million SNPs, verifying previously identified loci and identifying new variants in the *CPZ, MLF1,* and *RSRC1s* genes. Combining these data with additional proteomic analysis also identified variants in *NEFL* that predispose children to developing high-risk neuroblastoma [108]. Capasso et al. also further analyzed this 2101 patient cohort and found a functional variant in *CDKN1B* that was associated with neuroblastoma risk [109]. Evaluation of copy number variants between cases and controls furthermore identified variation at 1q21.1 in the *NBPF23* gene as another predisposing factor to neuroblastoma [110].

Recent efforts to better understand neuroblastoma tumor biology using next-generation sequencing of somatic tissue has also yielded new insights into predisposing germline variation from the peripheral blood. Several studies have reported a number of rare germline variants in neuroblastoma patients. The first such analysis by Pugh et al. found variants in *CHEK2*, *PINK1*, *PALB2*, and *BARD1* [82]. Subsequent studies identified pathogenic germline variation in *APC* and *BRCA2* [111] and *SMARCA4* [112]. Despite these interesting findings, the extent to which these rare germline variants contribute to the development of neuroblastoma has yet to be fully ascertained.

### 3.2. Predisposition to Neuroblastoma Genotypes

The entirety of the studies reported to this point evaluated predisposition to any type of neuroblastoma or more narrowly focusing on the development of low- or high-risk disease. A recent report by Hungate et al. tested the hypothesis that in addition to phenotypic risk group, germline susceptibility loci are also associated with neuroblastoma genotype. Specifically, the hypothesis was that the development of *MYCN*-amplification in neuroblastoma cells and these loci would be different than those that predisposed patients to *MYCN*-nonamplified high-risk neuroblastoma [113]. Approximately 50% of high-risk patients have *MYCN*-amplified tumors, and while all tumors classified as high-risk are clinically aggressive, it is well known that the biology of *MYCN*-amplified and *MYCN*-nonamplified high-risk neuroblastoma are disparate [114,115]. This study implemented a unique case/control distribution, comparing patients with *MYCN*-amplified tumors to patients who developed non-high-risk neuroblastoma and then evaluating these results in conjunction with a second analysis of patients with *MYCN*-nonamplified high-risk disease, compared to the same controls. In this analysis of over ten million SNPs, rs80059929, which is intronic to *KIF15*, was found to be significantly associated with the development of *MYCN*-amplified, high-risk disease and not with *MYCN*-nonamplified high-risk disease. Additionally, while previously reported variants in *BARD1* appeared to confer risk of developing high-risk disease in general, those in *LMO1* were only associated with the development of *MYCN*-nonamplified, high-risk tumors. These results highlight potential to uncover a deeper understanding of cancer biology by utilizing alternate approaches to genomic analysis.

Shortly after these results were published, Chang et al. evaluated 113 patients with high-risk disease and loss of 11q, a genomic feature common in *MYCN*-nonamplified patients with poor outcomes [1], comparing them to 5109 Caucasian controls [116]. Three SNPs, the most significant being rs10895322, were identified in *MMP20* that were not significant when comparing patients with *MYCN*-amplified tumors to the same controls. Loci at *BARD1* and *LMO1* were also associated with development of MYCN-nonamplified high-risk tumors, similar to previous analysis of this subgroup.

## 4. Future Directions

### 4.1. Germline Genetics Predisposing to Adverse Events

In addition to genetics predisposing children to developing neuroblastoma, additional efforts have uncovered whether germline genetics can also predispose patients to specific adverse events, such as treatment failure and the development of second cancers. Using Epstein–Barr Virus, immortalized lymphoblastoid cell lines treated with the cyclophosphamide derivative phosphoramide mustard, researchers identified two linked SNPs, rs9908694 and rs1453560, that were associated with resistance to therapy. Using a validation in a cohort of 2709 neuroblastoma patients, these SNPs were also significantly associated with decreased event-free survival (*p* = 0.01). rs9908694 is intronic to *IKZF3*, although the functional role of this variant remains to be elucidated. Additional studies are ongoing to determine if these or other SNPs are associated with survival in larger COG trials.

Epidemiologic studies have determined that neuroblastoma patients develop second malignant neoplasms (SMNs) at much higher rates than population controls [118]. Additionally, low-risk patients who received little to no chemotherapy or radiation also develop SMNs at increased rates, suggesting there may be additional genetic risk factors for this outcome beyond those described for conventional cytotoxic treatments [119]. Indeed, this hypothesis has been tested and validated in other tumor types [120]. To evaluate this question in neuroblastoma, data from the INRG Database were mined for patients who developed a second malignancy and had genotyping information to compare to similar patients who did not develop this outcome [121]. Using a candidate SNP approach, variants in two DNA repair genes *XRCC3* (rs861539) and *MSH2* (rs17036651) were identified as having the potential to predispose patients to developing second malignant neoplasms. However, these findings still require validation in larger cohorts.

### 4.2. Beyond Caucasians in Sporadic Neuroblastoma

To date, most GWAS in neuroblastoma has been restricted to patients of European decent. We do know, however, that African American and Native American patients are more likely to have high-risk neuroblastoma and worse event-free survival compared to Caucasians [122]. This suggests that different ethnic and racial groups are likely to have unique predisposing genetic factors [123,124]. Furthermore, deficiencies of understanding the differences in genetic predispositions between ethnicities can lead to unsuccessful applications of genomics in the clinic [125,126]. While some studies in neuroblastoma have used an African American cohort to validate findings from Caucasians [99,101,127], primary genome-wide analyses of race and ethnicities other than Caucasians have been sparse. This is in part because of the difficulties assembling a large non-Caucasian neuroblastoma cohort in the United States and, until recently, limited availability of haplotype blocks for non-Caucasians. Despite these challenges, Gamazon et al. performed a candidate SNP evaluation comparing 310 African American patients to 2709 neuroblastoma patients of European decent [117]. This analysis identified a novel variant in *SPAG16* that is significantly more common in African American children who developed high-risk neuroblastoma and rarely present in Caucasians. Furthermore, this variant was also associated with worse event-free survival (*p* = 0.007) and incorporation of this genotype into a Cox model of outcome negates the difference in survival previously observed between racial/ethnic groups. Additionally, a cohort of southern Chinese patients have been evaluated to validate specific neuroblastoma predisposition variants [128]. However, despite almost 1000 patients in the cohort, a true GWAS of this population has not been reported and risk variants specific to this population have yet to be identified. Overall predisposing factors to developing neuroblastoma in patients of non-European decent should be a priority for the field as these studies may find additional biologic features of this disease.

### 4.3. Collaboration with Therapeutic and Technological Advances

Insights into both familial and sporadic cases of neuroblastoma have led to an improved understanding of the fundamental biology that promotes neuroblastoma tumorigenesis. These discoveries continue to drive hypotheses for clinical translation and have generated avenues for targeted therapeutic development. Targeted therapies have been developed for ALK+ neuroblastoma and others are in development for many of the genetic aberrations described. In addition to the phase 3 trial evaluating crizotinib in newly diagnosed patients (NCT03126916), there is the recently closed phase 2 study combining crizotinib with conventional chemotherapy in pediatric solid tumors and anaplastic large-cell lymphoma (NCT01606878). Newer generations of ALK inhibitors such as ceritinib and lorlatinib, which can overcome resistance to crizotinib, are also under investigation (NCT03107988 and NCT01742286). Although these trials focus on children already diagnosed with neuroblastoma, it is also conceivable that children diagnosed with a germline ALK mutation early in life could benefit from regular screening for neuroblastoma and early initiation of an ALK inhibitor may be warranted.

Recognizing the need to direct more funds towards understanding genetic predispositions to pediatric diseases, the Gabriella Miller Kids First Research Act was signed in 2014. The law eliminates taxpayer dollars from financing presidential campaigns and party conventions, instead directing these funds into an approximately 126 million-dollar, 10-year pediatric research initiative through the National Institutes of Health (NIH). Since it launched in 2015, more than 18,000 samples from patients and their families have been collected and sequencing data is publicly available for more than 2000 patients in the database of Genotypes and Phenotypes (dbGaP). This fund is currently providing support to sequence 563 neuroblastoma patients and their parents. The impact of the Gabriella Miller Kids First Program is likely to be vast and represents another exciting collaborative environment to further our understanding of the genetic basis of pediatric cancer as a whole.

Massively paralleled genomic technologies, such as whole exome and whole genome sequencing, have improved and become more affordable, leading to massive amounts of data that are often siloed within institutions and difficult to compare between data sets. This is true not only for analysis of tumor tissue, but also for germline variance as demonstrated by the Gabriella Miller project. Issues of interoperability and data harmonization between studies have necessitated the recent development of data commons to house, merge, and serve these genomic data to researchers. To date, the largest such effort is the Genomic Data Commons (GDC), sponsored by the NIH and the National Cancer Institute (NCI). This data commons contains raw and processed somatic and germline genomic data linked to clinical and histological information from NCI-funded projects [129]. Neuroblastoma is fundamentally a rare disease, and pooling information in this manner allows researchers to have the sample sizes required to detect important biology from germline sequencing data [129].

While the GDC currently hosts data for neuroblastoma and other pediatric and adult tumors, neuroblastoma has long led the field in these types of efforts. The INRG database has been collating and harmonizing clinical data for almost a decade [6]. As the database has grown to over 20,000 neuroblastoma patients treated worldwide, efforts have been undertaken to link these rich clinical data to available genomic datasets. Germline genetics are available on a large subset of these patients allowing researchers to more readily link germline variation with clinical phenotype and genotype. Recognizing the importance of such efforts, additional pediatric oncology disease groups such as soft tissue sarcoma and germ cell tumors have begun to form their own data commons to better harness the power of combining genomic datasets. These efforts underscore the importance of data sharing within the scientific community to advance the care for children with neuroblastoma and identify those who may be predisposed to the disease.

## 5. Conclusions

Despite considerable advances in classification and treatment of neuroblastoma, our understanding of the genetic causes of neuroblastoma is still modest, and we struggle to clinically identify which children are at highest risk of the disease. While the full genetic underpinnings of neuroblastoma have yet to be fully elucidated, meaningful progress has been made. The development of new technologies and laboratory methods, combined with improved data sharing, is sure to deliver many more discoveries, allowing for an ever-greater understanding of the genetic complexity of the disease. Ultimately, this knowledge must be applied towards the goal of curing children with neuroblastoma with minimal therapeutic toxicity.

## Figures and Tables

**Table 1:** Heritable conditions that predispose patients to developing neuroblastoma.

Syndrome/Disease	Gene	Typical Genetic Alterations	Clinical Findings	Pre-Disposed Tumors
Congenital Central Hypoventilation Syndrome (CCHS) [32,33,36]	*PHOX2B*	Polyalanine and nonpolyalanine repeat expansion (frameshift or missense)	Respiratory dysfunction, autonomic dysfunction, Hirschsprung disease, neural crest tumors	Neuroblastoma, ganglioneuroma, ganglioneuroblastoma
ROHHAD [64,65,66,67]	Unknown	Unknown	Autonomic dysfunction, endocrinopathies, alveolar hypoventilation	Neuroblastoma, ganglioneuroma, ganglioneuroblastoma
Costello [58,59,60]	*HRAS*	Activating missense	Intellectual disability, coarse facial features, loose folds of skin, heart abnormalities, joint flexibility	Papilloma, rhabdomyosarcoma, neuroblastoma, transitional cell carcinoma
Noonan [52,53,54,60]	*PTPN11*, *SOS1*, *RAF1*, *KRAS*	Activating	Short stature, heat abnormalities, skeletal abnormalities, bleeding	Leukemia, neuroblastoma
Neurofibromatosis type 1 [55,56,57,60]	*NF1*	Activating	Abnormal skin pigmentation, neurofibromas, scoliosis	Neurofibroma, MPNST, brain tumors, leukemia, optic glioma, neuroblastoma
Beckwith–Wiedemann [68,69,70,71]	*CDKN1C* *, *H19*, *IGF2*, *KNBQOT1*	Abnormal methylation of chromosome 11 or uniparental disomy	Macrosomia, hemihypertrophy, abdominal wall defects, visceromegaly	Wilms tumor, hepatoblastoma, neuroblastoma
Li–Fraumeni [51,62,63]	*TP53*	Missense	Increased cancer risk	Breast cancer, osteosarcoma, brain tumors, leukemia, neuroblastoma, adrenocortical carcinoma, soft tissue sarcoma
Weaver Syndrome [72]	*EZH2*	Missense and truncating	Tall stature, intellectual disability, joint deformities, hypertelorism, micrognathia	Neuroblastoma
Familial Paraganglioma/Pheochromocytoma [73,74]	*SDHB* *, *SDHAF2*, *SDHC*, *SDHD*	Splice site, frameshift, nonsense	Growth of benign tumors in paraganglia	Paraganglioma, pheochromocytoma, neuroblastoma
Fanconi Anemia [75,76,77,78,79]	*FANCA*, *FANCC*, *FANCG*, *BRCA1*, *BRCA2* *, *PALB2* *, *BRIP1* *, and many others	Truncating, frameshift, missense	Bone marrow failure, organ defects, skeletal abnormalities	Leukemia, Wilms tumor, medulloblastoma, neuroblastoma, embryonal tumors, sarcomas, nephroblastoma

* mutations in this gene confer the greatest susceptibility to neuroblastoma in this syndrome. Abbreviations: MPNST, malignant peripheral nerve sheath tumor; CCHS, congenital hypoventilation syndrome; ROHHAD, rapid-onset obesity, hypothalamic dysfunction, hypoventilation and autonomic dysfunction.

**Table 2:** Germline variants that increase risk of developing neuroblastoma.

Candidate Gene(s)	Variant *	Genomic Location
*TP53* [63]	rs35850753	17p13.1
*CASC-15 and NBAT-1* [84]	rs6939340	6p22
*BARD1* [88]	rs6435862	2q35
*LMO1* [96]	rs2168101	11p15.4
*DUSP12* [100]	rs1027702	1q23.3
*DDX4* [100]	rs2619046	5q11.2
*IL31RA* [100]	rs10055201	5q11.2
*HSD17B12* [100]	rs11037575	11p11.2
*HACE1* [101]	rs4336470	6q16
*LIN28B* [101]	rs17065417	6q16
*CPZ* [106]	rs3796727	4p16
*MLF1* [106]	rs6441201	3q25
*NEFL* [108]	rs1059111	8q21
*CDKN1B* [109]	rs34330	12p13
*KIF15* [113]	rs80059929	3p21.31
*MMP20* [116]	rs10895322	11q22.2
*SPAG16* [117]	rs1033069	2q34

* Most significant single-nucleotide polymorphisms (SNPs).
